# The first crystal structure of the pyrrolo­[1,2-*c*]oxazole ring system

**DOI:** 10.1107/S2056989019011095

**Published:** 2019-08-23

**Authors:** Mohamed M. Zreigh, Harry Adams, Richard F. W. Jackson, Craig C. Robertson

**Affiliations:** a Department of Chemistry, Faculty of Science, Zawia University, PO Box 16168, Zawia, Libya; b Department of Chemistry, The University of Sheffield, Dainton Building, Sheffield S3 7HF, UK

**Keywords:** crystal structure, pyrrolo­[1,2-*c*]oxazole, hydrogen bonds

## Abstract

The title compound, C_7_H_4_F_3_NO_2_, 3-tri­fluoro­methyl-1*H*-pyrrolo­[1,2-*c*]oxazol-1-one, is the first crystal structure of the pyrrolo­[1,2-*c*]oxazole ring system: the fused ring system is almost planar (r.m.s. deviation = 0.006 Å). In the crystal, weak C—H⋯O and C—H⋯F hydrogen bonds link the mol­ecules into [001] chains and π–π stacking inter­actions consolidate the structure.

## Abstract 2

The title compound is the first crystal structure of the pyrrolo­[1,2-*c*]oxazole ring system. In the crystal, weak C—H⋯O and C—H⋯F hydrogen bonds link the mol­ecules into [001] chains and π–π stacking inter­actions consolidate the structure.

## Chemical context   

**Figure 1:**
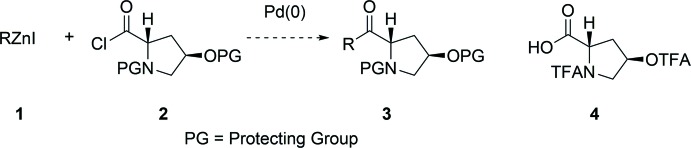
Proposed reaction scheme to access proline-derived ketones **3** from the Negishi coupling of organozinc reagents **1** with 4-hy­droxy­proline-derived acid chlorides **2**, specifically towards the formation of compound **3**

In the context of an approach to the synthesis of proline-derived ketones **3** by the proposed palladium-catalyzed Negishi coupling of organozinc reagent **1** with protected 4-hy­droxy­proline-derived acid chloride **2**, we needed access to a suitably *N*,*O*-diprotected 4-hy­droxy­proline derivative (Fig. 1 ▸). Our initial choice was to use TFA protection, since related cross-coupling reactions with the TFA-protected proline acid chloride had been successful (Deboves *et al.* 2001 ▸), and so the preparation of *N,O-*bis-tri­fluoro­acetyl-4-hy­droxy-l-proline **4** was attempted. The preparation of this compound had been reported, but without a detailed procedure (Mori *et al.*, 1986 ▸).

**Figure 2:**
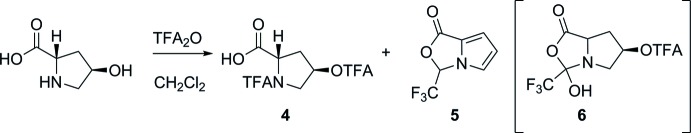
Reaction scheme for the synthesis of **5** along with the desired product **4**.

Treatment of (*2S*,4*R*)-4-hy­droxy­proline with tri­fluoro­acetic anhydride TFAA (3 eq.) in di­chloro­methane at 273 K, followed by heating at reflux, gave a mixture of two compounds, which could be separated by column chromatography (Fig. 2 ▸). The more polar compound was the desired *bis*-TFA protected (*2S*,4*R*)-4-hy­droxy­proline **4** (47%), and the less polar material was an unknown by-product **5** (52%). This latter unknown compound exhibited signals in the aromatic region of the ^1^H NMR spectrum, suggesting that the hy­droxy group had been eliminated and a pyrrole derivative had been formed. The mass spectrum obtained for **5** showed a base peak at *m*/*z* 190 (100%), and the IR spectrum exhibited a stretching frequency in the carbonyl region at 1781 cm^−1^. A crystal structure was obtained (see below), which confirmed that the compound was a new bicycle, a rare representative of the pyrrolo­[1,2-*c*]oxazole ring system as first described by Katritzky *et al.* (2004 ▸).

When the reaction was repeated under milder conditions, omitting the period of heating under reflux, the desired *bis*-TFA protected 4-hy­droxy-l-proline **4** was obtained in near qu­anti­tative yield, suggesting that it was partially converted into the novel pyrrolo­[1,2-*c*]oxazole **5** under reflux conditions, presumably by elimination from an inter­mediate of structure **6**.

## Structural commentary   

**Figure 3:**
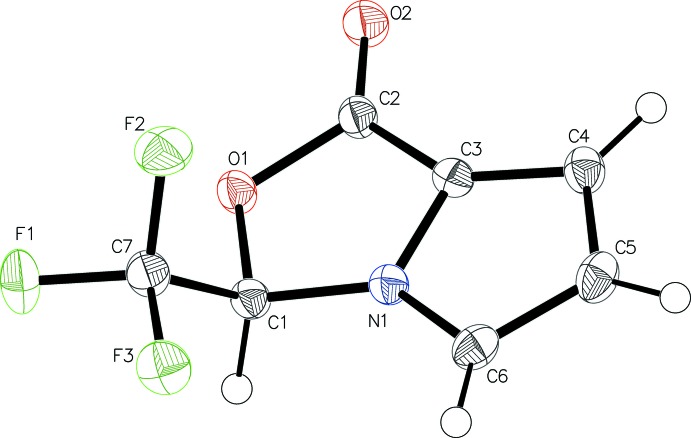
The mol­ecular structure of **5**, showing displacement ellipsoids drawn at the 50% probability level.

Compound **5** crystallizes in the monoclinic space group *P*2_1_/*c*: its asymmetric unit comprises of a single mol­ecule (Fig. 3 ▸). The fused bicyclic aromatic system is almost planar [r.m.s. deviation = 0.006 Å; dihedral angle between the five-membered rings = 0.86 (6)°]. Atom C7, which bears the fluorine atoms, is displaced from the ring plane by 1.282 (1) Å and F3 lies *anti* to O1 [O1—C1—C7—F3 = −176.33 (8)°]. In the arbitrarily chosen asymmetric mol­ecule, the stereogenic centre C1 has an *R* configuration but crystal symmetry generates a racemic mixture.

## Supra­molecular features   

**Figure 4:**
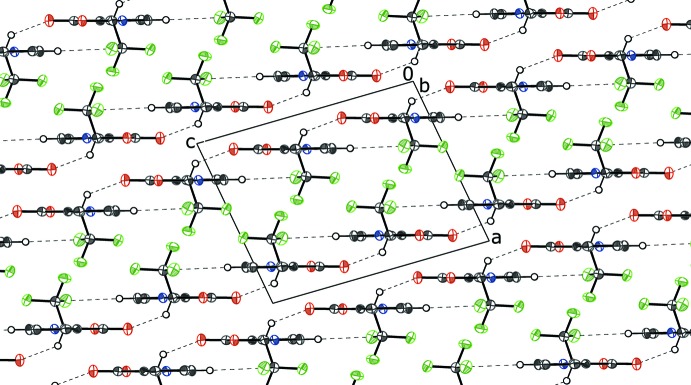
View along the *b* axis of the crystal packing for **5**, showing the alternating layers of inter­digitated CF_3_ groups and bicyclic ring systems. Hydrogen bonds are shown as dashed lines; hydrogen atoms not involved in forming these bonds are omitted for clarity.

In the crystal, two weak hydrogen bonds (Table 1 ▸) are observed between **5** and the adjacent mol­ecule related by the symmetry operation (*x*, −*y* + 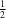, *z* − 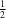). These form between the *sp* ^3^ hydrogen atom H1 and the carbonyl oxygen atom O2 and the aromatic proton H6 and F1 of the triflomethyl group: together, they generate an [001] chain. The mol­ecules pack in sheets parallel to the (010) plane with alternating layers of inter­digitated CF_3_ groups and π–π stacked ring systems (Fig. 4 ▸). The shortest π–π stacking inter­action between centrosymmetrically related N1/C3–C6 rings has a centroid–centroid separation of 3.5785 (7) Å with a vertical distance of 3.4196 (5) Å and a shift of 1.017 Å with an inter-planar angle of 0°.

## Database survey   

A search in the Cambridge Structural Database (CSD, V5.40, update February 2019; Groom *et al.*, 2016 ▸) was performed to confirm that there have been no previous crystal structures of the pyrrolo­[1,2-*c*]oxazole ring system.

## Synthesis and crystallization   

Tri­fluoro­acetic anhydride (0.33 ml, 2.31 mmol, 2.1 eq) was added dropwise to a stirred solution of *trans*-4-hy­droxy-l-proline (144 mg, 1.1 mmol) in CH_2_Cl_2_ (2 ml) at 273 K. The reaction mixture was warmed to room temperature, and then heated under reflux for 1.5 h. The excess of CH_2_Cl_2_ was removed under reduced pressure to give a crude product that was purified by column chromatography (petrol:ethyl acetate, 80:20%) to give 3-tri­fluoro­methyl-1*H*-pyrrolo­[1,2-*c*]oxazol-1-one (0.11 g, 52%) as a white powder, m.p. 338–340 K; *R*_f_ 0.27 (petrol:ethyl acetate, 80:20%); *v*_max_(film)/cm^−1^ 3150, 2977, 2918, 1781, 1548, 1318, 1276; δ_H_ (400 MHz; CDCl_3_) 6.20 (1H, *q*, *J* = 3.5), 6.62 (1H, *dd*, *J* = 2.5, 4.0), 6.91 (1H, *dd*, *J* = 1.0, 4.0), 7.12–7.15 (1H, m); δ_C_ (100 MHz; CDCl_3_) 111.2, 118.7, 119.2, 120.3 (CF_3_, *q*, *J* = 283.0), 121.7, 156.7. Analysis calculated for C_7_H_3_NO_2_F_3_: C, 43.9; H, 2.1; N, 7.3. Found: C, 43.92; H, 2.16; N, 7.10. *m*/*z* (ES^−^) 190 (*M* − H)^−^, 100%). Found: [*M* − H]^−^ 190.0115 C_7_H_3_NO_2_F_3_ requires 190.0116. Recrystallization from petroleum ether:ethyl acetate 80:20% solution led to colourless blocks of **5**.

The mass balance (47%) was the known *bis*-TFA-4-hy­droxy-l-proline **4** (Mori *et al.*, 1986 ▸).

## Refinement   

Crystal data, data collection and structure refinement details are summarized in Table 2 ▸. The H atoms were positioned geometrically (C—H = 0.93 Å for *sp* ^2^ aromatic and 0.98 Å for *sp* ^3^ methine CH atoms) and refined as riding atoms with relative isotropic displacement parameters *U*_iso_(H) = 1.2*U*_eq_ of the parent atoms.

## Supplementary Material

Crystal structure: contains datablock(s) I. DOI: 10.1107/S2056989019011095/hb7843sup1.cif

Structure factors: contains datablock(s) I. DOI: 10.1107/S2056989019011095/hb7843Isup2.hkl

Supporting information file. DOI: 10.1107/S2056989019011095/hb7843Isup3.cml

CCDC reference: 1919738

Additional supporting information:  crystallographic information; 3D view; checkCIF report

## Figures and Tables

**Table 1:** Hydrogen-bond geometry (Å, °)

*D*—H⋯*A*	*D*—H	H⋯*A*	*D*⋯*A*	*D*—H⋯*A*
C1—H1⋯O2^i^	0.98	2.46	3.2683 (13)	140
C6—H6⋯F1^i^	0.93	2.53	3.4065 (13)	156

Symmetry code: (i) 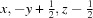.

**Table 2:** Experimental details

Crystal data	
Chemical formula	C_7_H_4_F_3_NO_2_
*M*_r_	191.11
Crystal system, space group	Monoclinic, *P*2_1_/*c*
Temperature (K)	100
*a*, *b*, *c* (Å)	8.2767 (5), 8.5106 (5), 10.5500 (7)
β (°)	99.443 (3)
*V* (Å^3^)	733.07 (8)
*Z*	4
Radiation type	Mo *K*α
μ (mm^−1^)	0.18
Crystal size (mm)	0.43 × 0.32 × 0.32

Data collection	
Diffractometer	Bruker APEXII CCD
Absorption correction	Multi-scan (*SADABS*; Bruker, 2009 ▸)
*T*_min_, *T*_max_	0.700, 0.746
No. of measured, independent and observed [*I* > 2σ(*I*)] reflections	13286, 1681, 1595
*R*_int_	0.033
(sin θ/λ)_max_ (Å^−1^)	0.650

Refinement	
*R*[*F* ^2^ > 2σ(*F* ^2^)], *wR*(*F* ^2^), *S*	0.030, 0.078, 1.08
No. of reflections	1681
No. of parameters	118
H-atom treatment	H-atom parameters constrained
Δρ_max_, Δρ_min_ (e Å^−3^)	0.39, −0.30

Computer programs: *APEX2* and *SAINT* (Bruker, 2009 ▸), *SHELXT* (Sheldrick, 2015*a* ▸), *SHELXL* (Sheldrick, 2015*b* ▸) and *OLEX2* (Dolomanov *et al.*, 2009 ▸).

## supplementary crystallographic information

### Crystal data

**Table:**

C_7_H_4_F_3_NO_2_	*F*(000) = 384
*M**_r_* = 191.11	*D*_x_ = 1.732 Mg m^−^^3^
Monoclinic, *P*2_1_/*c*	Mo *K*α radiation, λ = 0.71073 Å
*a* = 8.2767 (5) Å	Cell parameters from 9927 reflections
*b* = 8.5106 (5) Å	θ = 3.1–27.6°
*c* = 10.5500 (7) Å	µ = 0.18 mm^−^^1^
β = 99.443 (3)°	*T* = 100 K
*V* = 733.07 (8) Å^3^	Block, colourless
*Z* = 4	0.43 × 0.32 × 0.32 mm

### Data collection

**Table:**

Bruker APEXII CCD diffractometer	1595 reflections with *I* > 2σ(*I*)
φ and ω scans	*R*_int_ = 0.033
Absorption correction: multi-scan (SADABS; Bruker, 2009)	θ_max_ = 27.5°, θ_min_ = 2.5°
*T*_min_ = 0.700, *T*_max_ = 0.746	*h* = −10→10
13286 measured reflections	*k* = −11→11
1681 independent reflections	*l* = −13→13

### Refinement

**Table:**

Refinement on *F*^2^	0 restraints
Least-squares matrix: full	Hydrogen site location: inferred from neighbouring sites
*R*[*F*^2^ > 2σ(*F*^2^)] = 0.030	H-atom parameters constrained
*wR*(*F*^2^) = 0.078	*w* = 1/[σ^2^(*F*_o_^2^) + (0.0348*P*)^2^ + 0.3122*P*] where *P* = (*F*_o_^2^ + 2*F*_c_^2^)/3
*S* = 1.08	(Δ/σ)_max_ < 0.001
1681 reflections	Δρ_max_ = 0.39 e Å^−^^3^
118 parameters	Δρ_min_ = −0.30 e Å^−^^3^

### Special details

**Table:**

Geometry. All esds (except the esd in the dihedral angle between two l.s. planes) are estimated using the full covariance matrix. The cell esds are taken into account individually in the estimation of esds in distances, angles and torsion angles; correlations between esds in cell parameters are only used when they are defined by crystal symmetry. An approximate (isotropic) treatment of cell esds is used for estimating esds involving l.s. planes.

### Fractional atomic coordinates and isotropic or equivalent isotropic displacement parameters (Å^2^)

**Table:**

	*x*	*y*	*z*	*U*_iso_*/*U*_eq_	
N1	0.20468 (11)	0.42629 (11)	0.56351 (8)	0.0174 (2)	
O1	0.14217 (9)	0.27976 (9)	0.72729 (7)	0.01878 (18)	
O2	0.08840 (11)	0.47329 (9)	0.86114 (7)	0.02351 (19)	
C1	0.19006 (13)	0.26514 (12)	0.60398 (10)	0.0173 (2)	
H1	0.107086	0.208458	0.544158	0.021*	
C2	0.12893 (12)	0.43833 (12)	0.76072 (10)	0.0176 (2)	
C3	0.17020 (12)	0.52963 (12)	0.65500 (9)	0.0168 (2)	
C4	0.18460 (13)	0.68045 (13)	0.61032 (10)	0.0197 (2)	
H4	0.167818	0.773574	0.652411	0.024*	
C5	0.22998 (13)	0.66468 (14)	0.48775 (11)	0.0221 (2)	
H5	0.248941	0.747088	0.434193	0.027*	
C6	0.24167 (14)	0.50640 (14)	0.46028 (10)	0.0215 (2)	
H6	0.269396	0.463188	0.385775	0.026*	
C7	0.35527 (14)	0.18148 (13)	0.61947 (11)	0.0211 (2)	
F1	0.34284 (9)	0.03455 (8)	0.66179 (7)	0.03022 (19)	
F2	0.46888 (8)	0.25554 (9)	0.70256 (7)	0.03067 (19)	
F3	0.40810 (8)	0.17430 (9)	0.50653 (7)	0.02723 (18)	

### Atomic displacement parameters (Å^2^)

**Table:**

	*U*^11^	*U*^22^	*U*^33^	*U*^12^	*U*^13^	*U*^23^
N1	0.0210 (4)	0.0154 (4)	0.0160 (4)	−0.0006 (3)	0.0032 (3)	0.0002 (3)
O1	0.0248 (4)	0.0147 (4)	0.0178 (4)	−0.0008 (3)	0.0064 (3)	0.0003 (3)
O2	0.0321 (4)	0.0207 (4)	0.0193 (4)	0.0014 (3)	0.0089 (3)	−0.0002 (3)
C1	0.0204 (5)	0.0153 (5)	0.0163 (5)	−0.0018 (4)	0.0034 (4)	−0.0002 (4)
C2	0.0179 (5)	0.0149 (5)	0.0195 (5)	−0.0003 (4)	0.0019 (4)	0.0002 (4)
C3	0.0168 (5)	0.0167 (5)	0.0169 (5)	−0.0008 (4)	0.0023 (4)	−0.0014 (4)
C4	0.0180 (5)	0.0166 (5)	0.0243 (5)	−0.0009 (4)	0.0024 (4)	0.0014 (4)
C5	0.0201 (5)	0.0217 (5)	0.0243 (5)	−0.0020 (4)	0.0028 (4)	0.0071 (4)
C6	0.0242 (5)	0.0241 (5)	0.0168 (5)	−0.0007 (4)	0.0049 (4)	0.0039 (4)
C7	0.0229 (5)	0.0185 (5)	0.0221 (5)	0.0003 (4)	0.0044 (4)	0.0000 (4)
F1	0.0360 (4)	0.0185 (4)	0.0383 (4)	0.0071 (3)	0.0122 (3)	0.0059 (3)
F2	0.0220 (3)	0.0351 (4)	0.0319 (4)	0.0010 (3)	−0.0045 (3)	−0.0043 (3)
F3	0.0265 (4)	0.0300 (4)	0.0275 (4)	0.0021 (3)	0.0113 (3)	−0.0018 (3)

### Geometric parameters (Å, º)

**Table:**

N1—C1	1.4474 (13)	C3—C4	1.3792 (15)
N1—C3	1.3700 (13)	C4—H4	0.9300
N1—C6	1.3617 (14)	C4—C5	1.4108 (15)
O1—C1	1.4262 (12)	C5—H5	0.9300
O1—C2	1.4036 (13)	C5—C6	1.3846 (16)
O2—C2	1.2000 (13)	C6—H6	0.9300
C1—H1	0.9800	C7—F1	1.3374 (13)
C1—C7	1.5265 (15)	C7—F2	1.3339 (13)
C2—C3	1.4456 (14)	C7—F3	1.3363 (13)
			
C3—N1—C1	111.31 (8)	C3—C4—H4	127.0
C6—N1—C1	138.68 (9)	C3—C4—C5	106.00 (9)
C6—N1—C3	110.01 (9)	C5—C4—H4	127.0
C2—O1—C1	110.98 (8)	C4—C5—H5	125.6
N1—C1—H1	111.1	C6—C5—C4	108.82 (10)
N1—C1—C7	110.90 (9)	C6—C5—H5	125.6
O1—C1—N1	103.62 (8)	N1—C6—C5	106.68 (10)
O1—C1—H1	111.1	N1—C6—H6	126.7
O1—C1—C7	108.73 (8)	C5—C6—H6	126.7
C7—C1—H1	111.1	F1—C7—C1	110.80 (9)
O1—C2—C3	106.55 (8)	F2—C7—C1	111.87 (9)
O2—C2—O1	120.33 (9)	F2—C7—F1	107.88 (9)
O2—C2—C3	133.12 (10)	F2—C7—F3	108.00 (9)
N1—C3—C2	107.54 (9)	F3—C7—C1	110.14 (9)
N1—C3—C4	108.48 (9)	F3—C7—F1	108.01 (9)
C4—C3—C2	143.96 (10)		
			
N1—C1—C7—F1	177.53 (8)	C1—O1—C2—O2	179.38 (9)
N1—C1—C7—F2	57.11 (12)	C1—O1—C2—C3	−0.12 (11)
N1—C1—C7—F3	−63.02 (11)	C2—O1—C1—N1	−0.25 (11)
N1—C3—C4—C5	−0.21 (12)	C2—O1—C1—C7	117.77 (9)
O1—C1—C7—F1	64.22 (11)	C2—C3—C4—C5	−178.20 (14)
O1—C1—C7—F2	−56.21 (11)	C3—N1—C1—O1	0.55 (11)
O1—C1—C7—F3	−176.33 (8)	C3—N1—C1—C7	−115.95 (9)
O1—C2—C3—N1	0.46 (11)	C3—N1—C6—C5	−0.03 (12)
O1—C2—C3—C4	178.46 (14)	C3—C4—C5—C6	0.20 (13)
O2—C2—C3—N1	−178.94 (11)	C4—C5—C6—N1	−0.11 (13)
O2—C2—C3—C4	−0.9 (2)	C6—N1—C1—O1	−178.81 (12)
C1—N1—C3—C2	−0.64 (11)	C6—N1—C1—C7	64.69 (16)
C1—N1—C3—C4	−179.40 (8)	C6—N1—C3—C2	178.91 (9)
C1—N1—C6—C5	179.34 (11)	C6—N1—C3—C4	0.15 (12)

### Hydrogen-bond geometry (Å, º)

**Table:**

*D*—H···*A*	*D*—H	H···*A*	*D*···*A*	*D*—H···*A*
C1—H1···O2^i^	0.98	2.46	3.2683 (13)	140
C6—H6···F1^i^	0.93	2.53	3.4065 (13)	156

Symmetry code: (i) *x*, −*y*+1/2, *z*−1/2.
